# 387. High Rates of Bacteremia and Fluoroquinolone Resistance during an Outbreak of Shigellosis among People Experiencing Homelessness and Opioid Use Disorder in Philadelphia, PA

**DOI:** 10.1093/ofid/ofae631.122

**Published:** 2025-01-29

**Authors:** Eleanor Stedman, Andrea Molin, Valencia Oglesby, Erin Torpey, Stephanie Spivack, Sara K Schultz

**Affiliations:** Temple University Hospital, Philadelphia, PA; Temple University Hospital, Philadelphia, PA; Temple University Hospital, Philadelphia, PA; Temple University Hospital, Philadelphia, PA; Temple University Health System, Philadelphia, Pennsylvania; Temple University Hospital, Philadelphia, PA

## Abstract

**Background:**

In October 2023, the city of Philadelphia, PA reported a dramatic rise in shigellosis cases from *Shigella flexneri*, leading to widespread diarrheal illness and many hospitalizations. Notable qualities of this outbreak included high antibiotic resistance, high rates of bacteremia in hospitalized patients, a death due to invasive shigellosis, and extensive housing insecurity and opioid use disorder (OUD) among those affected.

**Figure:**
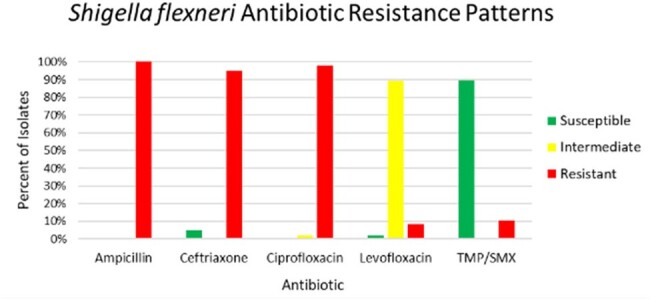

Rates of antibiotic susceptibility and resistance among tested isolates of *Shigella flexneri*.

**Methods:**

All patients hospitalized at Temple University Health Systems (TUHS) with confirmed shigellosis between October 2023 and April 2024 were included in the study. Diagnosis was made via stool culture, blood culture, and/or polymerase chain reaction (PCR) stool pathogen panel. Charts were reviewed for patient age, sex, housing status, history of OUD, stool and blood culture data, stool pathogen panel data, antibiotic susceptibility data, and any death due to shigellosis.

Hospitalizations and Bacteremia Over Time

**Figure:**
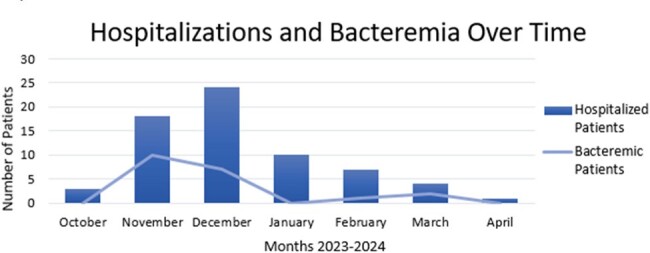

Number of patients hospitalized and number of patients with documented Shigella flexneri bacteremia per month during an outbreak of shigellosis.

**Results:**

Sixty-seven patients were hospitalized at TUHS from October 2023 to April 2024 with confirmed shigellosis. The average age of patients was 43 and 60% were male. Bacteremia was documented in 21 patients (31.3%). Among cultured isolates, 0% were ampicillin or ciprofloxacin susceptible, 2.1% were levofloxacin susceptible, 4.8% were ceftriaxone susceptible, and 89.6% were trimethoprim-sulfamethoxazole (TMP-SMX) susceptible. One patient died from shigellosis, a 1.5% mortality rate in hospitalized patients. Housing insecurity was documented for 67% of patients, and 68.7% of patients had OUD.

**Conclusion:**

This shigellosis outbreak in Philadelphia saw an unprecedented rate of bacteremia among hospitalized patients. Cultured isolates were highly beta-lactam and fluoroquinolone resistant. TMP-SMX was the only oral antibiotic with reliable susceptibility. Most hospitalized patients had housing insecurity and OUD. Due to the fecal-oral route of infection, outbreak containment challenges reflect the insufficient sanitation facilities for people experiencing homelessness.

**Disclosures:**

**Sara K. Schultz, MD FACP FIDSA**, AbbVie: Advisor/Consultant

